# Membrane interaction and structure of the transmembrane domain of influenza hemagglutinin and its fusion peptide complex

**DOI:** 10.1186/1741-7007-6-2

**Published:** 2008-01-15

**Authors:** Ding-Kwo Chang, Shu-Fang Cheng, Eric Aseen B Kantchev, Chi-Hui Lin, Yu-Tsan Liu

**Affiliations:** 1 Institute of Chemistry, Academia Sinica, Taipei, Taiwan 11529, Republic of China; 2 Institute of Bioengineering and Nanotechnology, 138669, Singapore

## Abstract

**Background:**

To study the organization and interaction with the fusion domain (or fusion peptide, FP) of the transmembrane domain (TMD) of influenza virus envelope glycoprotein for its role in membrane fusion which is also essential in the cellular trafficking of biomolecules and sperm-egg fusion.

**Results:**

The fluorescence and gel electrophoresis experiments revealed a tight self-assembly of TMD in the model membrane. A weak but non-random interaction between TMD and FP in the membrane was found. In the complex, the central TMD oligomer was packed by FP in an antiparallel fashion. FP insertion into the membrane was altered by binding to TMD. An infrared study exhibited an enhanced membrane perturbation by the complex formation. A model was built to illustrate the role of TMD in the late stages of influenza virus-mediated membrane fusion reaction.

**Conclusion:**

The TMD oligomer anchors the fusion protein in the membrane with minimal destabilization to the membrane. Upon associating with FP, the complex exerts a synergistic effect on the membrane perturbation. This effect is likely to contribute to the complete membrane fusion during the late phase of fusion protein-induced fusion cascade. The results presented in the work characterize the nature of the interaction of TMD with the membrane and TMD in a complex with FP in the steps leading to pore initiation and dilation during virus-induced fusion. Our data and proposed fusion model highlight the key role of TMD-FP interaction and have implications on the fusion reaction mediated by other type I viral fusion proteins. Understanding the molecular mechanism of membrane fusion may assist in the design of anti-viral drugs.

## Background

Influenza hemagglutinin (HA) is responsible for the attachment and fusion of the virus to the target membrane. Mature HA is composed of HA1 (attachment) and HA2 (fusion) subunits connected by a disulfide linkage. HA2 can be divided into the fusion peptide (FP) domain, the heptad repeat (HR) regions, transmembrane domain (TMD) and the cytoplasmic tail (CT). The functional roles of FP and HR domains have been demonstrated rather clearly [1-4]: the hydrophobic FP domain is sequestered in the resting state but exposed and inserted into the target membrane on low pH activation; the HR domain undergoes extensive refolding to form the hairpin structure to bring the two membranes proximal and probably provides free energy to overcome the barrier of membrane merger. A previous study by Lai et al. [5] revealed that the functional fusion peptide of influenza virus had a kinked helix structure with a fixed angle in the micellar environment. However, the role played by TMD remains controversial except for the recognition that it anchors the fusion protein on the viral membrane and is involved in the late stages of the fusion process. As evidence for the latter proposition, cells expressing a glycosylphosphatidylinositol (GPI)-anchored ectodomain of HA have been shown to support hemifusion to target membranes at low pH [6], implying a TMD role in transiting membrane hemifusion to full fusion. The result was corroborated by a stringent TMD length requirement for supporting full membrane fusion [7], strongly suggesting that it is necessary for TMD to span both inner and outer leaflets to fulfill its function of driving complete fusion via hemifusion. On the other hand, a mutational study of the HIV-1 TMD demonstrated that substitution of one specific residue in TMD did not alter the fusion protein function, whereas replacement of TMD with that of CD4 [8] or of vesicular stomatitis virus G [9] abolished the viral fusion activity without affecting transport and cleavage properties.

The structure, orientation and interaction of the TMD of HA2 (X:31 strain) has been investigated by Tatulian and Tamm [10]. It was found that the highly helical TMD inserted into lipid bilayer nearly perpendicular to the membrane surface, probably forming oligomers of various sizes and water-accessible pores. They suggested that TMD had a role at the late stages of membrane fusion, including dehydration of water at the apposing membrane surfaces. Melikyan et al. [11] have shown that substitution of the TMD of HA (Japan) with TMD from other unrelated proteins does not affect membrane fusion. On the other hand, mutation of selected residues within TMD abolished fusion [7].

Taken together, these findings led to the hypothesis that there may be not an absolute sequence-specific requirement for TMD to interact with FP in the fusion reaction [7,12].

As a widely held model on protein-induced fusion proposes that the ectodomain of fusion proteins consists of heptad repeat domains sandwiched between FP and TMD capable of forming a helix hairpin, it is of interest to explore whether there exists any interaction between FP and TMD and, if so, what is the nature of the interaction and its involvement in the fusion process. In addition, to clarify the architecture of TMD in the membrane in complex with FP, we conducted biophysical experiments on the peptides derived from HA2 TMD and FP in a model membrane. Owing to the potentially weak interaction between TMD and FP in the membranous environment, fluorescence spectrophotometry was employed which is most suitable for long-range (>10 Å) interactions in lieu of the nuclear magnetic resonance (NMR) measurements that are sensitive to short-range association (<5 Å). We found that TMD self-associated more tightly than FP in the membrane. The two peptide molecules form a loose complex in an antiparallel manner, with TMD oligomers interspersed with FP molecules and modulation of lipid penetration of FP by interacting with TMD.

An operational model of the fusion mechanism based on the findings of the present work and previous study was constructed to shed light on the role of TMD and FP with an emphasis on the promotion of the transition from hemifusion to full fusion by the two regions in HA2 represented by TMD and FP.

Elucidation of the function of TMD and FP and their interaction in the context of HA-induced membrane fusion may provide a missing piece in the mechanistic study of a host of cell-cell and cell-virus fusion events in which the helix-bundle was shown to be the core structure, for example, in fusion mediated by other proteins involved in the intracellular vesicle fusion [13-15].

## Results

### Influenza TMD peptide associates with and inserts into the membrane

**Figure 1:**
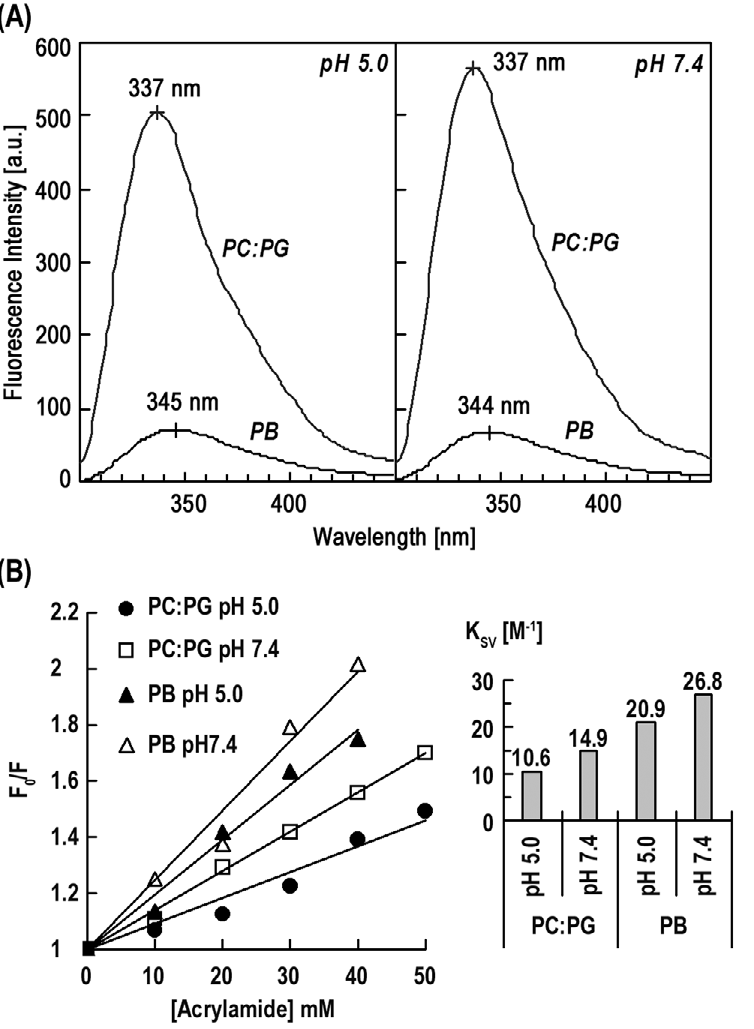
**Hemagglutinin TMD peptide inserts into membrane bilayer at acidic and neutral pH**. (A) The blue shift and enhancement of fluorescence intensity of tryptophan residues in TMD when incubated in DMPC:DMPG vesicles attest to the location of TMD in the membrane hydrophobic milieu. The emission maximum for tryptophan in an aqueous environment is 350 nm. (B) *K*_SV _acrylamide quenching measurements also indicate deep insertion of TMD into the membrane interior. The dramatic decrease in *K*_SV _in the vesicular dispersion compared with that in PB buffer shows that tryptophan side chains are embedded deep into the membrane. Moreover, a twofold reduction in *K*_SV_, as well as decreased *K*_SV _on neutralization, upon incubating in PC:PG vesicles at pH 5.0 compared with that at pH 7.4 suggests that the TMD penetration is deeper at acidic pH.

The membrane association can be determined by the intensity and blue shift of tryptophan residues in the TMD peptide in the hydrophobic milieu of membrane bilayer. In Figure 1 we show Trp fluorescence intensity changes upon mixing with DMPC:DMPG (1:1 molar ratio) vesicles (Figure 1A) and the Stern-Volmer constant (*K*_SV_) obtained from quenching with acrylamide (Figure 1B). A shift of emission maximum from 345 to 337 nm and the enhancement of emission as the peptide in aqueous buffer was added to the vesicular dispersion indicate the immersion of the TMD peptide in the lipid bilayer. Insertion into the membrane is ascertained by a marked decrease in *K*_SV_, from 20.9 to 10.6 at pH 5.0 and from 26.8 to 14.9 at pH 7.4, for TMD in association with vesicles. The smaller *K*_SV _value, 10.6, compared with 14.9 at pH 7.4 coupled with the difference in the ratio of *K*_SV _in the two pH tested suggests that the insertion of the peptide is deeper at acidic than neutral pH.

### Self-assembly of TMD in the membrane bilayer can be deduced from Rhodamine self-quenching by variation of composition of the fluorescent-labeled peptide

**Figure 2:**
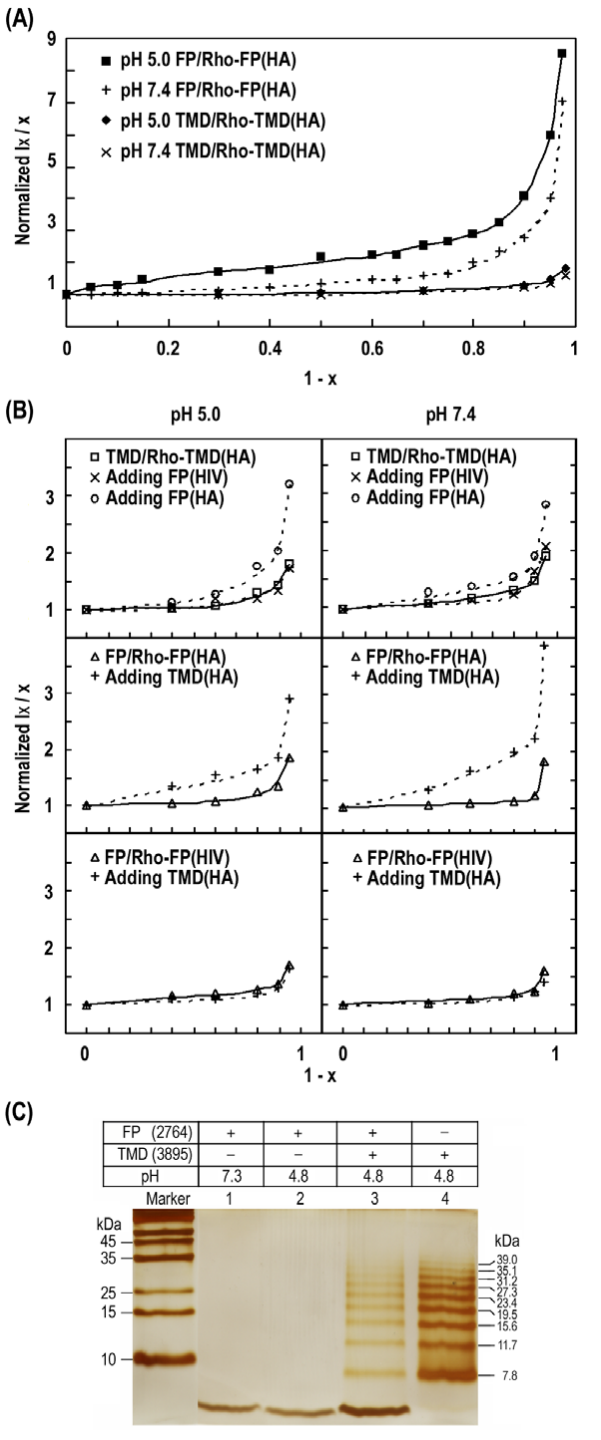
**Rhodamine composition experiments detect tight self-association of TMD and non-random interaction of TMD:FP association**. (A) The large self-quenching (i.e. low intensity) of Rhodamine is virtually unchanged in the *x *= 0.3–1.0 region as the labeled TMD manifests packing of TMD molecules into a tight subunit in the membrane at pH 5.0 and 7.4. In contrast, labeled FP exhibits less self-quenching, indicative of a loose association for the peptide molecules. (B) Association between TMD and FP in the bilayer is not arbitrary as FP of HIV-1 gp41 causes no change in Rho-TMD dequenching or Rho-FP of gp41 dequenching was not affected by mixing with TMD. Change in Rho-FP or Rho-TMD of HA2, in contrast, is obvious when complexed to their counterpart. Note that the smallest value of *x *in the measurements is 0.02 for (A) and 0.05 for (B). (C) A higher propensity of self-association for TMD than FP is revealed by SDS-PAGE. Lanes 1 and 2 show that FP has less tendency than TMD to form oligomers in SDS in either neutral or acidic buffer. In contrast, TMD formed multiple oligomeric species (lane 4) at pH 4.8 for which minimal association owing to disulfide linkage is expected. The association between TMD and FP is not strong enough to sustain the dispersing force of SDS detergent and the electric field as seen in lane 3.

We have probed the self-assembly of influenza HA2 FP in the membrane and found a loose association for the FP molecules [16]. In the present study, the dependence of Rhodamine (Rho) self-quenching on the composition of the fluorescence label was used to probe the self-association of TMD (Figure 2A) and TMD-FP association (Figure 2B). In Figure 2A, the following two observations are noteworthy for both acidic and neutral pH: first, the flatness of the normalized intensity in the *x *= 1–0.3 range (which reflects mainly short-range intra-subunit interaction), compared with HA2 FP [16] (where *x *denotes the fraction of labeled peptide), suggests a very tight TMD association; second, in the low *x *(<0.3) regime (which emphasizes long-range inter-subunit interaction) a much smaller increase in the normalized intensity than the case of FP also corroborates the idea of tight self-binding for HA2 TMD molecules. This conclusion is further supported by the association between FP and TMD described in the next section. Another line of evidence for tighter self-association of TMD is provided by sodium dodecyl sulfate polyacrylamide gel electrophoresis (SDS PAGE) measurements (Figure 2C). The multiple oligomeric species of TMD at low pH is in marked contrast with the single monomer band for FP, demonstrating higher propensity of self-association for TMD. Moreover, the pattern for the mixture of TMD and FP is the combination of the individual TMD and FP bands, indicating that the interaction between the two peptides cannot sustain the dispersing force exerted by the SDS detergent and the underlying electric field. The data are in line with the idea of weak interaction between TMD and FP, as is further elaborated in the following sections. Oligomerization of TMD has been documented by Tatulian and Tamm [10].

The nature of binding between TMD and FP was revealed in Figure 2B. For both pH levels tested, the normalized intensity of the Rho dye labeled to TMD or FP of HA2 increases on mixing with their counterparts, while no change is observed when FP of human immunodeficiency virus is added to the TMD-containing solution. This result clearly indicates that the interaction between TMD and FP is not random. We also found that the intensity enhancement is less pronounced for the labeled TMD than the labeled FP when the TMD:FP complex is formed, indicating that self-packing of TMD is tighter and TMD likely forms the inner core in the complex. The difference is easily detected in top and middle panels of Figure 2B for pH 7.4 in which Rho-TMD experiences less dequenching than Rho-FP at the same *x *value; also, at smaller *x*, Rho-FP shows a larger increase suggesting a dispersed FP subunit by complexing to TMD (i.e. reduced intra-subunit association), supporting the concept of an inner TMD core for the TMD:FP complex which is more directly shown in the next section.

### Rhodamine self-quenching measurements reveal association of TMD with FP and TMD probably forms the inner core in the membrane

Using the Rhodamine group attached to HA2 FP and TMD peptides to compare the effect of complex formation on the self-quenching allows determination of the configuration of the FP:TMD complex in the membrane. In the experiments, the fluorescence intensity as a percentage of that in the presence of triton X-100 (for complete dequenching) is used as a gage for aggregation, with smaller values representing tighter association. As summarized in Table 1, a substantial increase in Rho-labeled FP at low pH upon addition of TMD suggests association of these two peptides. This indicates that, at pH 5.0, FP molecules self-assemble with considerable strength, but are broken up upon incubating with TMD in the bilayer. (At neutral pH, dequenching of Rho-FP by TMD addition is not as pronounced owing to a rather loose self-assembly.) It is likely that FP monomers are wrapped on the exterior of the TMD oligomer, as deduced from the markedly smaller intensity of Rho labeled to TMD than that labeled to FP. Alternatively, the loosely associated FP oligomers, as evidenced by much larger Rho-FP dequenching than that of TMD displayed in Table 1, may distribute around the tight-packed TMD. Either interpretation is in line with the previous conclusion of weak self-association of HA2-FP in the membrane [16]. A negative control is provided by the self-quenching data on mixing of HA2 TMD and gp41 FP of HIV-1 (which also forms a loose self-assembly as shown in Table 1 and by Kliger et al. [17]); no detectable change in the extent of quenching is observed compared with gp41 FP alone (Table 1).

**Table 1:** Assembly of TMD and TMD:FP complex of HA as probed by the Rhodamine self-quenching. The FP peptide of HIV gp41 was used as a negative control. Values are expressed as a percentage of the Rhodamine intensity in the presence of 0.2% Triton X-100.

A	B	pH	A	A+B	Difference
Rho-TMD (HA)	FP (HA)	5.0	8.7	9.3	+0.6
		7.4	12.9	13.0	+0.1
Rho-TMD (HA)	FP (HIV)	5.0	10.4	11.3	+0.9
		7.4	12.1	13.0	+0.9
Rho-FP (HA)	TMD (HA)	5.0	35.5	55.9	+20.4
		7.4	63.1	70.4	+7.3
Rho-FP (HIV)	TMD (HA)	5.0	63.2	63.1	-0.1
		7.4	79.5	77.9	-0.4

It is noteworthy that a substantial Rho-TMD dequenching upon addition of FP is observed in the composition variation study, particularly at low *x *values (Figure 2B) while little dequenching is found for Rho-TMD complexed to FP. Conceivably, the long-range interaction between the fluorescent labels attached to TMD, which is monitored in the low *x *regime (Figure 2B), is affected by FP addition; however, the short-range interaction probed by experiments leading to data in Table 1 using fully labeled TMD exhibits little change with FP addition, indicating a very compact TMD oligomer (possibly trimer) subunit un-dissociable by complexing to FP.

Again the association for both FP and TMD in the complex is tighter at acidic pH than neutral pH (Table 1).

### FRET measurements between NBD and Rhodamine afford evidence for interaction between TMD and FP

**Figure 3:**
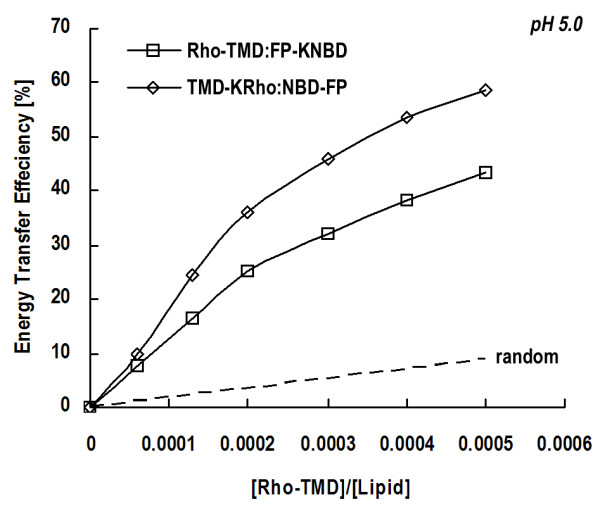
**NBD-Rho FRET efficiency as a function of acceptor concentration**. NBD (donor) and Rhodamine (acceptor) were labeled at the ends of FP and TMD peptides, respectively, to examine interaction between the two molecules. Different combinations are depicted by various curves as indicated and the dashed curve is derived from random distribution of *R*_0 _= 60 Å donor-acceptor pair [36]. Higher FRET efficiency from experimental data for the labeled NBD-Rho pair than that from the theoretical computation at any given Rhodamine concentration suggests association between TMD and FP in the membrane bilayer.

The interaction between TMD and FP can be most directly investigated by FRET experiments using NBD and Rho labeled to the two peptides as the donor-acceptor pair. Figure 3 displays FRET efficiency measured at pH 5.0 versus acceptor concentration. The higher efficiency obtained for experimental curves than that calculated with random distribution of the two peptides in the lipid clearly indicates an interaction between them.

### FP molecules are arranged in antiparallel orientation in the TMD:FP complex

**Figure 4:**
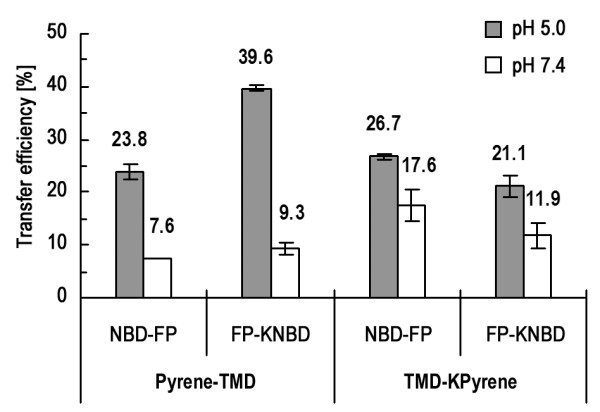
**FRET measurements disclose interaction between TMD and FP in an antiparallel manner**. The efficiency of FRET between pyrene and NBD labeled to the N- and C-termini of TMD and FP peptides in different combinations is compared to determine the orientation of the TMD:FP complex. FRET efficiency is larger for the donor and acceptor fluorophores attached to the opposite ends of TMD and FP. It is also noted that the interaction between FP and TMD is stronger at pH 5.0 than at 7.4 as reflected by greater transfer efficiency.

The association between FP and TMD prompted us to investigate the orientation of FP with respect to TMD in the complex. FRET experiments were conducted because of its sensitivity to the distance between the donor and acceptor fluorophores. To differentiate between these two possible orientations, we labeled donors and acceptors at each of the two ends of the peptides and compared the differential FRET efficiency. Figure 4 shows the pyrene to NBD fluorescence energy transfer efficiency monitored at the pyrene emission peak (380 nm). It is clear that FRET is larger when the donor-acceptor pair is labeled at different ends than FRET for the pair attached to the same end at either N- or C-terminus of the peptides. The pattern is the same at both pH 5.0 and 7.4, with larger FRET efficiency at acidic pH suggesting a stronger FP:TMD complex at the fusogenic pH.

### Insertion depth of HA2 FP is altered by the interaction with TMD

**Figure 5:**
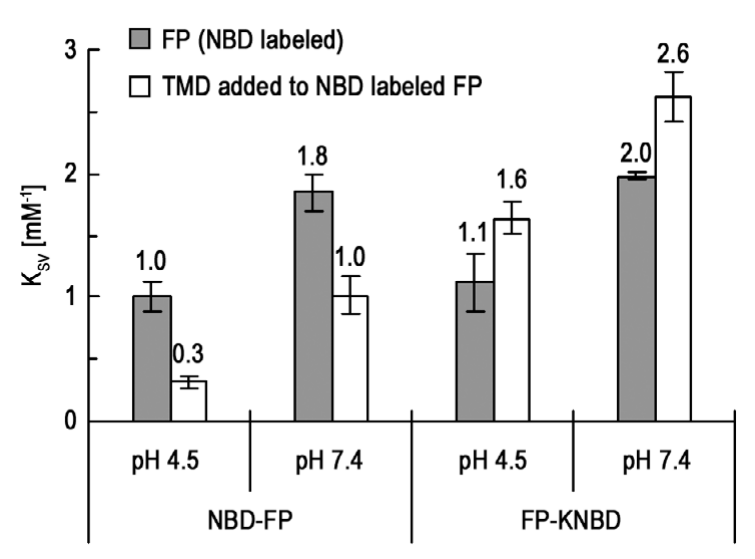
**FP inserts deeper into the membrane on association with TMD as probed by *K*_SV _values measured from NBD quenched by Co^2+^**. The increased *K*_SV _values of NBD labeled to the C-terminus of FP and the decrease in *K*_SV _for NBD N-terminally labeled to FP when interacting with TMD can be rationalized by a better alignment of FP on complexing to TMD. The results also support the notion of FP-TMD interaction in the membrane.

It has been shown by Tatulian and Tamm [10] that TMD inserted into the membrane nearly perpendicular to the membrane surface. On the other hand, HA2 FP has been found to insert obliquely into the membrane. Hence, it is of interest to examine the effect of TMD:FP formation on the membrane insertion depth and angle of FP. As illustrated in Figure 5, *K*_SV _of cobalt quenching of NBD labeled at the N-terminus of FP decreases with the introduction of TMD. In stark contrast, *K*_SV _increases upon complexing to TMD for NBD labeled at the C-terminus of FP. The effect of adding TMD on *K*_SV _is the same for pH 5.0 and 7.4. The data strongly suggest that the N-terminal portion of FP penetrates deeper while the C-terminus shallower as the TMD:FP complex forms in the membrane. Importantly, as discussed in the following, the alteration of the insertion depth of the N- and C-termini of FP upon complex formation leads to the idea that FP aligned more parallel to TMD with its N-terminus close to the C-terminus of TMD. The finding may have a bearing on the role of TMD in promoting membrane hemifusion to complete fusion transition, as is elaborated in the Discussion. Compatible with the previous results [18], insertion of FP is deeper at acidic pH.

### The Tb^3+^/DPA measurements suggest that the HA2 TMD peptide does not exhibit membrane leakage activity as FP does

**Figure 6:**
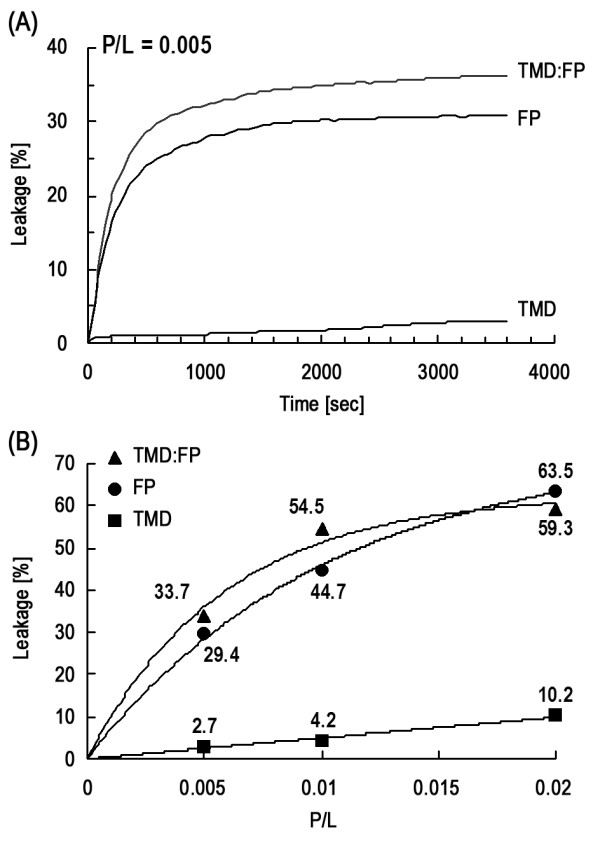
**Demonstration of the lack of membrane leakage activity of TMD in comparison with FP**. (A) Membrane leakage experiments using Tb^3+^/DPA assay to monitor membrane activity of TMD, FP and TMD:FP complex. Both FP and FP:TMD display dose-dependent leakage activity whereas TMD alone exhibits little activity. It is noted that the characteristic time of leakage is approximately 200 s for *P*/*L *= 0.05. (B) Profile of the steady-state leakage versus *P*/*L *for FP, TMD and FP:TMD. (*P*/*L *is the peptide to lipid ratio.)

Figure 6 demonstrates the lack of membrane leakage activity of TMD in comparison with FP. Thus, for TMD in POPC vesicular suspension at pH 7.4, little leakage of encapsulated Tb^3+ ^is observed and the extent of leakage is insignificantly different for TMD:FP complex and FP, indicative of low leakage activity for TMD and no enhancement of the activity of FP when complexed to TMD. It is of interest to note that TMD:FP or FP molecules are able to disrupt the membrane at neutral pH, implying that the pH-dependence of the influenza HA2 resides mainly at or prior to the stage of helix hairpin formation [19].

### HA2 TMD inserts into membrane nearly perpendicularly and promotes dehydration but causes less membrane perturbation than FP as revealed by ATR-FTIR measurements

**Figure 7:**
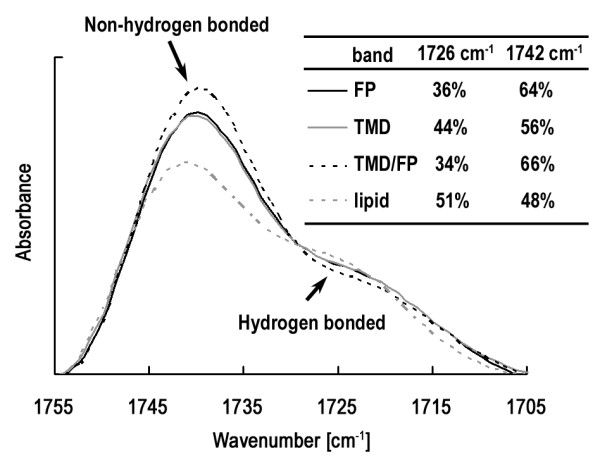
**The ATR-FTIR absorption bands of amide carbonyl vibration of DMPC:DMPG lipid alone and in the presence of TMD, FP and their complex**. The higher-frequency band is assigned to the non-hydrogen bonded lipid owing to dehydration, while the lower-frequency band is assigned to hydrated hydrogen bonded lipid. It is seen that the percentage of dehydrated bands increases as the two peptides form a complex and FP has a higher dehydration level than TMD.

To examine the membrane interaction of TMD, and membrane perturbation of TMD alone and TMD:FP complex, infrared experiments were carried out. The secondary structure and orientation of TMD, FP and TMD:FP are summarized in Table 2. Helix accounted for 64% of the secondary structure for TMD, in qualitative agreement with the values obtained by Tatulian and Tamm [10]. No significant change in helix content was observed for TMD:FP complex, whose helicity is approximately an average of that of TMD and FP. The insertion angle for TMD was found to be 34° with respect to the normal of the membrane, slightly larger than the value reported by Tatulian and Tamm [10]. Similar to the helix content, the insertion angle of TMD:FP helix is an average of that of TMD and FP. We also note in Figure 7 that the extent of dehydration is greater for FP than TMD. Moreover, the membrane perturbation probed by the change in lipid acyl chain orientation caused by FP and by TMD (Table 2) revealed that FP has a greater effect than TMD. The lesser membrane-perturbing effect of TMD than FP seen here is compatible with the results of leakage experiments (Figure 6). The smaller insertion angle for TMD than that for FP and less dehydration of TMD may be correlated with its smaller perturbation on the membrane acyl chain orientation. Importantly, as shown in the inset of Figure 7, the dehydration caused by TMD:FP is more pronounced than FP and TMD individually, indicating a synergetic membrane-perturbing effect of the formation of TMD:FP complex suggesting a role of TMD and FP association in destabilizing the fusing membranes.

**Table 2:** The secondary structure and orientation of helix, beta sheet and lipid acyl chain of FP, TMD and FP/TMD 1:1 complex in DMPC:DMPG 1:1 vesicular solution with *L*/*P *= 50 at pH 5.0. Values were obtained by averaging three or four sets of data.

	Lipid	FP	TMD	Complex
*Secondary structure*				
α-helix (%)		26 ± 3	64 ± 1	46 ± 7
β-sheet (%)		55 ± 5	20 ± 2	28 ± 1
Unordered (%)		9 ± 3	---	9 ± 8
β-turns (%)		11 ± 3	17 ± 4	17 ± 3
*Helix axis orientation*				
*R*^ATR^		1.82 ± 0.01	2.46 ± 0.22	2.07 ± 0.08
*Θ *(°)		60 ± 1	34 ± 1	49 ± 2
*Beta-strand orientation*				
*R*^ATR^		1.18 ± 0.10	N.A.	1.28 ± 0.20
*Φ *(°)		56 ± 5	N.A.	52 ± 7
*Acyl chain tilt angle*				
*R*^ATR^	1.09 ± 0.09	1.78 ± 0.45	1.31 ± 0.04	1.62 ± 0.12
*δ *(°)	27 ± 4	49 ± 12	36 ± 1	45 ± 4

## Discussion

### TMD of HA2 inserts into membrane bilayer with a pH-dependent depth

We have shown that HA2 FP penetrated more deeply into the membrane at low pH. The result in Figure 1 on TMD membrane-insertion depth displays similar pH dependence. The deeper insertion at acidic pH for both TMD and FP, as discussed in the following, may have ramifications for the low-pH activation of HA2-mediated fusion process.

### Self-assembly of TMD is stronger than FP and is insignificantly affected by the incorporation of FP

A previous investigation revealed loose self-association of FP in the membrane [16]. Here we show in Table 1 that TMD molecules form tightly packed oligomeric subunits in the membrane which are tighter than FP as deduced from the greater Rhodamine self-quenching for TMD. No discernible dequenching is observed for Rhodamine-labeled TMD as FP is added, while Rhodamine conjugated to FP has enhanced dequenching with TMD incorporation. This suggests that tight TMD packing is intact upon interacting with FP whereas inter-FP distance becomes longer for loosely aggregated FP monomers when attracted by tightly associated TMD oligomers nearby. Another line of evidence for a more stable oligomer formed by TMD can be visualized in Figure 2C, in which only the monomeric FP band is displayed. More indirect evidence for tighter association of TMD than FP and that TMD constitutes the inner core of the TMD:FP complex can be deduced from Figure 2B. The association between the two kinds of molecules is further affirmed by the FRET results shown in Figure 3 indicating larger transfer efficiency than random distribution of the two peptides from NBD to Rhodamine conjugated, respectively, to TMD and FP at the opposite ends. The orientation between TMD and FP can be resolved by FRET experiments in which pyrene (donor) and NBD (acceptor) were labeled to TMD and FP at either N- or C-terminus (Figure 4). The result clearly showed an antiparallel TMD:FP association.

### Membrane interaction of TMD and FP

It is interesting that, unlike FP, TMD displays little membrane disrupting effect despite the closer TMD packing, as shown in the leakage experiments summarized in Figures 6A and 6B. This is corroborated by the ATR-FTIR data on the dehydration (Figure 7) and lipid acyl chain orientation (Table 2). This could be explained by the smaller membrane insertion angle (closer to membrane normal) for TMD, causing less membrane perturbation. After all, TMD serves as an anchor for the viral fusion protein and therefore should not induce membrane permeation and death of the virus.

The membrane perturbing effect of TMD and FP has also been studied by ATR-IR measurements as shown in Figure 7. The larger fraction of carbonyl vibrational peak for the TMD:FP complex than that for either TMD or FP reveals a synergistic membrane-perturbing effect of the TMD:FP complex. As membrane dehydration represents a major barrier to fusion, this result suggests that association of the two HA2 domains, primarily by perturbing the membrane bilayer at the fusing site, promotes membrane merger mediated by the influenza hemagglutinin.

In this work, fluorophotometry, such as FRET and Rhodamine self-quenching, was used to study the association between TMD and FP and membrane organization of TMD. It turns out that this is appropriate because the active distance for these fluorescence measurements is in the range of 10–50 Å, which covers the loose interaction between FP and TMD. The loose TMD:FP complex inferred from the present work is in line with the sodium dodecyl sulfate gel electrophoresis experiment in which the two coincubated peptides exhibited separate TMD and FP bands under the electric field and dispersing force of SDS (Figure 2C).

### Biological implication of FP:TMD interaction

**Figure 8:**
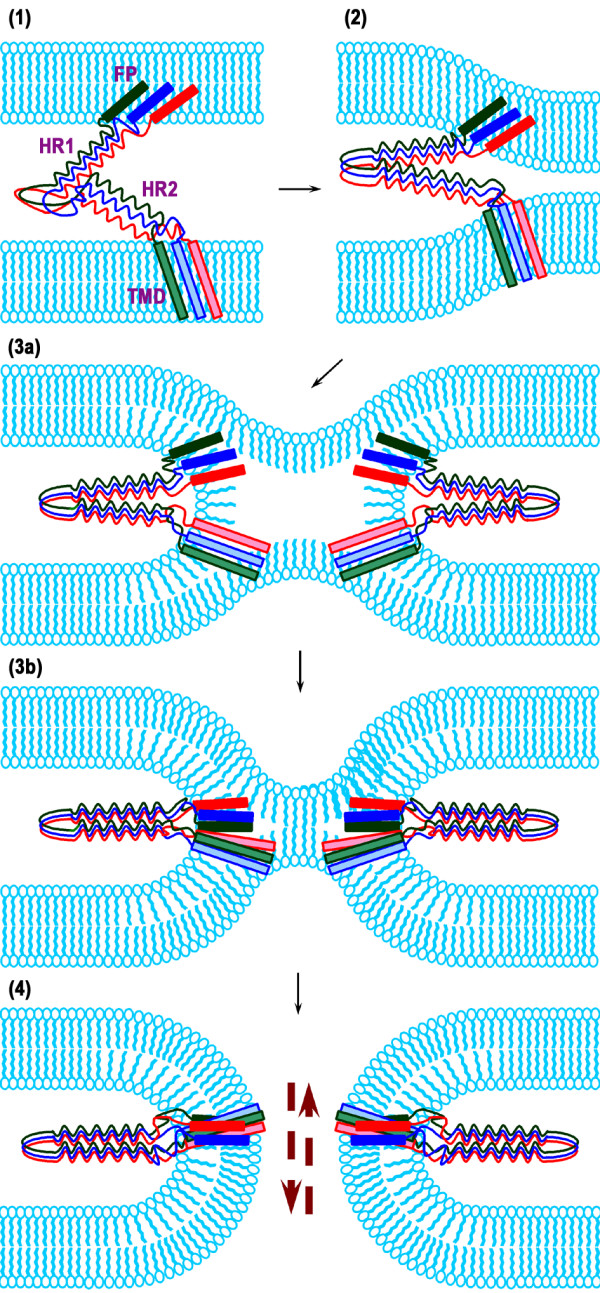
**Schematic illustration on the role of FP and TMD in the late stages of HA2-mediated fusion**. (1) In the pre-hairpin stage, FP inserts into the target membrane following disengagement of HA1 from HA2. The inner leaflet of the bilayer is minimally disrupted by FP with an oblique insertion angle. Note the loose FP self-assembly and tight self-association of TMD in the membrane. (2) Low pH-induced refolding of HR1 and HR2 regions of the HA2 driven by strong interactions between them. The two apposing membranes are pulled in proximity and bulged-out to facilitate the merge. (3) Driven by the energy liberated by HR1-HR2 association and additional force provided by the polar, conformationally plastic linker segment downstream of FP and the membranetropic pre-TM region, the two fusing membranes undergo dehydration, deformation and coalescence of the outer leaflets, causing hemifusion. In the process, the compact TMD homo-trimer approaches the loose FP aggregate and may be interspersed with FP molecules, gradually forming the TMD-FP complex, which is not specific per se, with TMD in the inner core. Nonetheless, the interaction is sufficiently strong to align FP with TMD to a certain extent and deepen FP penetration into the inner leaflet, further destabilizing the bilayer. (4) Partly as a result of the complex formation-enhanced perturbation of both leaflets of the effector and target membranes, the hemifusion diaphragm transits to an inceptive fusion pore, concomitant with the six-helix bundle formation of HR1 and HR2. By this stage, the recruitment of adjacent TMD:FP triplex subunits cooperatively stabilizes the initial pore and its dilation to facilitate the mixing of cytoplasmic contents.

As elaborated above, it is possible that the TMD oligomers are surrounded by FP on the external surface or loosely associated FP molecules disperse around TMD homo-oligomers. It has been shown that the polar segment immediately following FP of HIV-1 gp41 is conformationally plastic [12,20] and that the tryptophan-rich pre-TM stretch possesses membrane activity [21]. Given the involvement of TMD in the hemifusion-to-complete fusion transition and the stringent length requirement for this function [7] we propose a working model for the late steps of HA-mediated fusion (Figure 8). At the pre-hairpin stage, FP inserts into the target membrane; trimerization is mainly mediated through self-association of the HR1 region while HR2 domain is somewhat unordered. Perhaps owing to the flexibility and membrane activity of the FP-proximal region and the membrane-perturbing pre-TM region [22], refolding of the pre-hairpin structure occurs when HA is exposed to acidic pH, pulling the two apposing membranes close. In the membrane interior, FP and TMD move towards each other in antiparallel orientation to form a loose complex, with self-assembled TMD surrounded by FP or interspersed with FP in a somewhat straggling manner, in view of the report that fusion activity is retained with the TMD segment replaced by TMD from other membrane proteins [8,9]. In addition to deepening the FP penetration into the lipid bilayer and further deforming the membrane at the fusing site, the loose interaction between TMD and FP may foster clustering of neighboring FP and the associated TMD molecules, a necessary step for the fusion pore formation and enlargement. We propose that the latter process constitutes a major step for FP and TMD to exert their function. Concomitantly, HR1 and HR2 of the ectodomain form a helix hairpin bundle in the space between the apposing membranes. The free energy released from the rearrangement and conformational change enables the fusion protein and viral and target membranes to surmount the barrier of membrane dehydration and deformation (destabilization) required for membrane coalescence [23]. In other words, the synergetic membrane-perturbing effect (Figure 7) and the deepening membrane penetration of FP resulting from complexing to TMD (Figure 5), in combination with TMD traversing both leaflets of bilayer, eventually cause the rupture of the inner leaflets of both attending membranes resulting in full fusion by the cooperative FP:TMD cluster recruited to the fusion site.

We have provided several lines of evidence for the loose association between TMD and FP in the model membrane, in contrast to highly specific recognition of the receptor by the surface subunit of the viral fusion protein. Perhaps the role of TMD in the membrane fusion is twofold: first, mechanically it anchors the fusion protein onto the viral membrane and secures the oligomerization of the fusion protein through its tight self-association, and importantly, it does not destabilize the membrane in the absence of FP; second, it has a weak interaction with FP, thereby reinforcing the destabilizing effect of FP on the inner leaflet of the target membrane by deepening FP membrane insertion (Figure 7). This latter effect is manifested by the requirement of TMD length for different phenotypes of fusion, hemifusion and full-fusion activity [7], because spanning both leaflets of the bilayer for TMD is conceivably a prerequisite for TMD to execute this function. The differential results on the effect of altering the basic residue in the middle of the HIV-1 TMD sequence [9,24] may be related to the weak association between TMD and FP deduced herein (Figure 2C). The concept that the role of TMD in the fusion process lies more in disrupting the inner leaflet of the fusing membranes than the specific interaction with FP is consistent with the inability of a GPI-anchored HA ectodomain to mediate full fusion [6].

## Conclusion

The results presented in the work highlight the importance of the interaction of TMD with the membrane and TMD in complex with FP in the steps leading to pore initiation and dilation and shed some light on the fusion reaction mediated by other type I viral fusion proteins.

## Methods

The DMPC, DMPG and POPC used in this work were obtained from Avanti Polar Lipids (Alabaster, AL, USA), acrylamide, NBD and Triton X-100 from Sigma (St. Louis, MO, USA) and 5(6)-carboxytetramethylrhodamine hydrochloride (TAMRA) from Molecular Probes, Inc. (MPI, Eugene, OR, USA). Terbium chloride hexahydrate (TbCl_3_) and 2,6-pyridinedicarboxylic acid (DPA) were purchased from Acros Organics (Geel, Belgium). All reagents were used without further purification.

The peptides of TMD (GYKDWILWISFAISCFLLCVVLLGFIMWACQRG) and FP (GLFGAIAGFIENGWEGMIDGWYGFR) of HA2 (strain X:31) of influenza virus were synthesized using a Fmoc/t-Bu solid-phase method on a Rainin PS3 peptide synthesizer (Protein Technologies, Tucson, AZ, USA). Labeling of TAMRA or NBD, purification and characterization of the peptides used were described previously [25,26]. The pyrene labeling protocol was detailed in Additional file 1. Lys was added at the end of the sequence while the fluorescent probes were labeled on the C-terminus.

Small unilamellar vesicles (SUVs) were prepared by solubilizing DMPC:DMPG mixture (1:1) in chloroform:methanol (4:1, v/v). The lipidic solution was dried under a stream of nitrogen until a thin film was obtained and then dried using a centrifuge under vacuum overnight to ensure the movement of all solvent. The phospholipid was resuspended in PB buffer and sonicated for 30 min with a Sonicor (New York, NY, USA) ultrasonic processor.

### Fluorescence spectrophotometry

All fluorescence experiments were performed on a Hitachi F-2500 Fluorescence Spectrophotometer at 37°C, unless indicated otherwise. A scan rate of 300 nm/min was used in the wavelength scan measurements.

#### Acrylamide quenching experiments

The fluorescence quenching study monitors the accessibility of Trp to the acrylamide quencher. Thus, a larger quenching constant of Trp by the aqueous phase quencher acrylamide indicates that the Trp is located closer to the membrane interface. Fluorescence emission spectra in the 300–450 nm range were recorded by using a 280 nm excitation wavelength with a cutoff filter at 300 nm. The slit bandwidths of excitation and emission were 5 and 2.5 nm, respectively. An incremental amount of acrylamide stock solution (1 M) was added to the 1 μM TMD peptide solutions (in PB buffer or in DMPC:DMPG 50:50 μM) to make final concentration of acrylamide up to 50 mM. Appropriate blanks were subtracted to obtain the corrected spectra and corrections owing to dilution were made to the observed fluorescence intensities. The data were analyzed using the Stern-Volmer equation [27]:

*F*_0_/*F *= 1 + *K*_SV_·[*Q*]

where *F*_0 _is the fluorescence intensity at the zero quencher concentration, *F *is the fluorescence intensity at any given quencher concentration [*Q*], whereas *K*_SV _represents the apparent Stern-Volmer quenching constant, obtained from the slope of the plot of *F*_0_/*F *versus [*Q*].

#### Rho-labeled/unlabeled peptide composition experiments

In the experiments on the composition variation of Rho-labeled peptide, the fraction of labeled peptide, *x*, was varied from 0.02 or 0.05 to 1. For self-association measurements of HA2 TMD or FP, the concentrations were kept at 1 μM/100 μM/100 μM of peptide/DMPC/DMPG. To investigate the association between TMD and FP of HA2 or HIV, a total concentration of 0.06 μM of each peptide (labeled and unlabeled) in DMPC:DMPG 30 μM:30 μM was used. Excitation and emission wavelengths of 530 and 578 nm, respectively, were used with slit bandwidth of excitation and emission of 10 nm. The normalized emission intensity *I*_*x*_/*x *was plotted against 1 - *x *[28].

It is noted that intra-trimeric interaction is detected for *x *values near 1 since nearly all peptide molecules are labeled and, therefore, quenching arises predominantly from the close neighbors within the same trimer. In contrast, for low *x *values, the probability of finding a pair of labeled peptides is slim and hence quenching arises mainly from labeled peptides in nearby trimers.

#### Association tendency of TMD and FP by Rho fluorophore

The Rho self-quenching experiments were carried out to examine the propensity of association of TMD with FP. To DMPC:DMPG (30/30 μM) vesicles at pH 5.0 or 7.4, the Rho-labeled TMD (or FP) was added followed by adding the unlabeled FP (or TMD). We used 0.06 μM of each peptide and the parameters were the same as those used in the Rho composition experiments described above. The 100% reference intensity was taken from the fluorescence measured in the peptide/lipid dispersion solubilized with 0.2% (v/v) Triton X-100.

#### FRET between Rho-labeled TMD peptide and NBD-labeled FP

The Förster distance (*R*_0_), at which the FRET efficiency is 50%, of the NBD-Rho pair (donor-acceptor) is about 56 Å [29]. NBD and Rho were labeled on FP and TMD peptides, respectively, at either N- or C-terminal end. The FRET between NBD and Rho was measured at 50°C by adding Rho-TMD to NBD-FP/DMPC/DMPG 0.06:150:150 μM. The ratios of [Rho-TMD]/[NBD-FP] were 0.3, 0.6, 1, 1.5, 2 and 2.5. To investigate the changes of NBD intensity, the excitation and the emission wavelengths were set at 467 and 530 nm, respectively, with a response of 0.04 s and slit bandwidth of excitation and emission of 10 nm.

To calculate the FRET efficiency, the intensity of donor (NBD-FP) without acceptor (Rho-TMD) was taken as 100%:

Efficiency (%) = *I*^donor+acceptor^/*I*^donor ^× 100

where *I*^donor+acceptor ^and *I*^donor ^are the intensities of NBD-FP/Rho-TMD mixture and NBD-FP only, respectively.

#### FRET between Pyrene-labeled TMD peptide and NBD-labeled FP

The measurements of FRET from Pyrene to NBD were recorded to investigate the alignment between TMD and FP peptides. The Förster distance *R*_0 _of the pyrene-NBD pair (donor-acceptor) is about 33 Å [29]. TMD and FP peptides were labeled by pyrene and NBD, respectively, on either N-terminus or C-terminus. Pyrene-labeled TMD was added to the DMPC:DMPG (15:15 μM) vesicular solution followed by the addition of the same amount of NBD-labeled FP. The final concentration of each peptide was 0.06 μM. To monitor the pyrene probe, the excitation and the emission wavelengths were set at 344 and 380 nm, respectively, with slit bandwidth of excitation and emission of 10 nm.

FRET efficiency is calculated according to (2) except that *I*^donor+acceptor ^and *I*^donor ^are the intensities of pyrene-TMD/NBD-FP mixture and pyrene-TMD only, respectively.

#### Co^2+ ^quenched NBD

NBD fluorescence can be quenched by divalent cobalt ions [30] via a collisional quenching mechanism. Similar to acrylamide quenching of Trp, a large quenching constant by the aqueous cation reflects a closer proximity of NBD tag to the membrane interface. For Co^2+ ^quenching experiments, the fluorescence of NBD-FP with/without TMD peptide in DMPC:DMPG 15:15 μM vesicles at pH 5.0 or 7.4 was measured until the intensity attained a steady value. The final concentration of each peptide was 0.06 μM. An incremental amount of CoCl_2 _stock solution (0.1 M) was then injected into the cuvette to give final Co^2+ ^concentration in the range 0.04–2.0 mM. Corrections owing to dilution were made to the observed fluorescence intensities. All parameters were the same as those used for NBD-Rho FRET experiments and the data were analyzed using (1).

#### Tb^3+^/DPA leakage experiments

The method is based on the enhancement of the lanthanide metal Tb^3+ ^fluorescence when the aromatic chelator DPA is liganded to the ion. Large unilamellar vesicles (LUVs) of POPC containing Tb^3+ ^were prepared as described previously [19,31,32].

To quantitate the extent of leakage observed in the Tb^3+^/DPA assay, FP or TMD peptide or TMD:FP 1:1 complex were added to a solution containing 40 μM POPC/Tb^3+^, 50 μM DPA, 100 mM NaCl, 10 mM Tris at pH 7.4. The fluorescence was recorded at ambient temperature with excitation and emission wavelengths of 270 and 490 nm, respectively, and 10 nm bandwidth for both excitation and emission. The percentage leakage of Tb^3+ ^was calculated as follows:

Leakage (%) = [(*F*_t _- *F*_0_)/(*F*_max _- *F*_0_)] × 100

where *F*_max _is obtained by adding 0.05% (v/v) Triton X-100 and *F*_0 _is equivalent to the values for DMSO controls.

### SDS-Polyacrylamide gel electrophoresis (SDS-PAGE)

HA2 FP and TMD peptides were dissolved in HFIP and mixed with lysoPC (1-dodecyl-2-hydroxyphosphatidylcholine) in ethanol as described by Tatulian and Tamm [10]. The organic solvents were removed under a stream of nitrogen followed by high vacuum for 1 h. The dried mixtures were then resuspended in either neutral (43 mM imidazole, 35 mM HEPES, pH 7.3) or acidic buffer (80 mM GABA, 20 mM acetic acid, pH 4.8) and sonicated for 6 min before mixing with the Laemmli buffer (pH 6.8) composed of 62.5 mM Tris-HCl, 25% glceryol, 2% SDS and 0.01% Bromophenol Blue. The concentrations of peptide and lysoPC were around 0.5 and 3 mM, respectively, and the pH of the acidic buffer mixed with Laemmli buffer was raised to about 5.3. For each lane of sample loading, 5 μl of the peptide/lysoPC was mixed with 10 μl Laemmli buffer, except that in lane 3, 5 μl of each of the TMD and FP in lysoPC were mixed before added to 20 μl Laemmli buffer. The molecular weight of each peptide is indicated in parentheses in Figure 2C. Electrophoresis was conducted at 20 mA constant current for 90 min. The image shows the peptide migration in 18% separating gel with 0.1% SDS (pH 8.8). FP exhibits less tendency than TMD to form oligomers in SDS in either neutral or acidic buffer, as shown in lanes 1 and 2. In contrast, TMD formed multiple oligomeric species (lane 4). The association between TMD and FP is not strong enough to counter the dispersing force of SDS detergent and the electric field gradient as seen in lane 3.

### ATR-FTIR measurements

Each of the tested peptides was homogenized in a small quantity of HFIP and incubated in pH 5.0 PBS-buffered SUVs to make a final *L*/*P *= 50. The sample was subsequently spread on the germanium surface until solvent had evaporated completely. The ATR sample covered with a homemade box was kept in full D_2_O hydration (D_2_O/lipid ratio >35 based on the ratio of absorbance peaks of D-O/C-H stretching).

ATR-FTIR spectra were recorded on a BIO-RAD FTS-60A spectrometer equipped with a KBr beamsplitter and a liquid nitrogen-cooled MCT detector. The incoming radiation was polarized with a germanium single diamond polarizer (Harrick, Ossining, NY, USA). The 45° germanium ATR-plate (2 mm × 5 mm × 50 mm) was cleaned using a plasma cleaner (Harrick) before depositing the sample. After 300 scans at a spectral resolution of 2 cm^-1^, the data were smoothed with triangular apodization and the absorption peaks were analyzed using the Peakfit program to obtain the secondary structure components [33].

The infrared linear dichroic ratio is defined by *R*^ATR ^= *A*_∥_/*A*_⊥ _[34,35], where *A*_∥ _and *A*_⊥ _are the absorbances at parallel and perpendicular polarizations of the incident infrared light, respectively. The tilt angles, relative to the membrane director, of lipid acyl chain (*δ*), α-helix molecular axis direction (*θ*) and β-strand axis (*Φ*) were calculated from equations described in Additional file 1.

## Abbreviations

ATR, attenuated total reflectance; DMPC, 1,2-dimyristoyl-sn-glycero-3-phosphocholine; DMPG, 1,2-dimyristoyl sn-glycero-3-phosphoglycerol; FP, fusion peptide; FRET, fluorescence resonance energy transfer; HA, hemagglutinin; HR, heptad repeat; lysoPC, 1-dodecyl-2-hydroxyphosphatidylcholine; NBD, 4-chloro-7-nitrobenz-2-oxa-1,3-diazole; *P*/*L*, peptide to lipid ratio; POPC, 1-palmitoyl-2-oleoyl-sn-glycero-3-phosphatidylcholine; Rho, Rhodamine; SDS PAGE, sodium dodecyl sulfate polyacrylamide gel electrophoresis; TMD, transmembrane domain.

## Authors' contributions

DKC designed the experiments and wrote the manuscript; SFC carried out the fluorescence experiments; EABK synthesized labeled and unlabeled peptides; CHL performed gel electrophoresis measurement; YTL executed the infrared study.

## Supplementary Material

Additional file 1 This additional file give details of the pyrene labeling, supplemental figures and references. Click here for file
