# Supplementary material for: Membrane interaction and structure of the transmembrane domain of influenza hemagglutinin and its fusion peptide complex
Source: BMC Biol. 2008 Jan 15;6:2. doi: 10.1186/1741-7007-6-2 (PMC2267159; doi:10.1186/1741-7007-6-2)
Supplement: Additional file 1 — This additional file give details of the pyrene labeling, supplemental figures and references. [file 1741-7007-6-2-S1.doc]

**Supplemental material**

***Pyrene labeling***

To the peptide resin (5 μmol) suspended in DMF (2 mL), a solution of 1-pyrenebutanoic acid (14 mg, 50μmol) and PyBOP (29 mg, 55 μmol) in DMF cotaining 5 vol % N-methylmorpholine (0.50 mL) were added and the mixture agitated with a stream of N_2_ over 10 h. The resin was washed with DMF and dichloromethane, the peptide was cleaved using the standard protocol and the crude was dissolved in 1,1,1,3,3,3-hexafluoro-2-propanol (HFIP). After removal of the solvent, pure peptide was obtained after semipreparative RP-HPLC (Vydac C18 column)using H_2_O-ACN gradients (containing 0.1 vol % TFA).

***Calculating the tilt angle (θ) of α-helix inserting into membranes relative to the membrane director and the tilt angle (δ) of lipid acyl chain***

ATR interface electric field amplitudes are given by (Harrick, 1967):

$$E_{\text{x}}=\frac{\text{2cos}\gamma\sqrt{\sin^{2}\gamma-n_{21}^{2}}}{\sqrt{1-n_{21}^{2}}\sqrt{\left(1+n_{21}^{2}\right)\sin^{2}\gamma-n_{21}^{2}}}$$

$$E_{y}=\frac{2\cos\gamma}{\sqrt{1-n_{21}^{2}}}$$

$$E_{z}=\frac{2\cos\gamma\sin\gamma}{\sqrt{1-n_{21}^{2}}\sqrt{\left(1+n_{21}^{2}\right)\sin^{2}\gamma-n_{21}^{2}}}$$

where *E_x_*, *E_y_*, and *E_z_* represent the electric field amplitudes along the x, y, z axes. *n*_1_ and *n*_2_ are the refractive indices of germanium and a thick film on germanium surface, respectively; *n*_21_ = *n*_2_/*n*_1_; and *γ* is the angle of incidence between the beam and the normal to the crystal surface, where *n*_1_=4, *n*_2_=1.43, and *γ* =45°. ATR interface electric field amplitudes used are according to the two-phase model (Harrick, 1967; Tamm and Tatulian, 1997).

The infrared linear dichroic ratio is defined by :

$$R^{ATR}=A_{//}/A_{\perp}=\frac{\left\langle M_{x}^{2}\right\rangle E_{x}^{2}+\left\langle M_{z}^{2}\right\rangle E_{z}^{2}}{\left\langle M_{y}^{2}\right\rangle E_{y}^{2}}$$

where *A*// and *A*_⊥_ are the absorbances for polarized parallel and perpendicular radiation, respectively. *M_x_*, *M_y_*, and *M_z_* are the transition dipole moments along the x, y, z axes, and a bracket denotes a time and space average over all transition dipole moment during the characteristic time of the IR experiment.

Order parameters, *S_Helix_* and *S_L_* that describe the orientation distribution of the α-helix structure and of the lipid hydrocarbon chains, respectively, are given by (Tamm and Tatulian, 1993):

$$S_{\text{Helix}}=\text{1/f}_{\text{Helix}}\left(\frac{2}{3\cos^{2}\alpha-1}\right)\left(\frac{E_{x}^{2}-R_{1655}^{\text{ATR}}E_{y}^{2}+E_{z}^{2}}{E_{x}^{2}-R_{1655}^{\text{ATR}}E_{y}^{2}-2E_{z}^{2}}\right)$$

$$S_{\text{L}}=-2\frac{E_{x}^{2}-R_{2920}^{\text{ATR}}E_{y}^{2}+E_{Z}^{2}}{E_{x}^{2}-R_{2920}^{\text{ATR}}E_{y}^{2}-2E_{z}^{2}}$$

where $\text{R}_{1655}^{\text{ATR}}$ and $\text{R}_{2920}^{\text{ATR}}$ indicate the dichroic ratio at 1655 cm^-1^ and 2920 cm^-1^, respectively, *f_Helix_* is the fraction of amino acid residues in α-helical conformation and *α* is the angle of the orientation of the transition moment of the vibration relative to the molecular director. *α *=38° is taken for calculating *S_Helix_* (Marsh et al., 2000) and* α *=90° for *S_L_*.

The angle between the membrane director and the α-helix molecular axis direction (*θ*) or lipid acyl chain (*δ*) is related to *S_Helix_*, *S_L_* by:

$$S_{\text{Helix}}=\left(\frac{3\left\langle\cos^{2}\theta\right\rangle-1}{2}\right)$$

$$S_{\text{L}}=\left(\frac{3\left\langle\cos^{2}\delta\right\rangle-1}{2}\right)$$

***Calculating tilt angle*** *(Φ)* ***of β-strands inserting into membranes relative to the membrane director***

The orientation of β-strands could be derived from amide I band at 1628 cm^-1^. Assuming that the orientation of the strand within the sheet is not changed on hydration or fluidization of the lipid membranes, the changes in the orientation of the sheets may be determined from the dichroic ratios of the amide I band alone (Marsh 1997):

$$\text{R}_{1628}^{\text{ATR}}=\frac{\text{E}_{\text{x}}^{2}}{\text{E}_{\text{y}}^{2}}+\frac{2\left\langle\text{cos}^{2}\zeta\right\rangle\left\langle\text{sin}^{2}\beta\right\rangle}{\text{1 - }\left\langle\text{cos}^{2}\zeta\right\rangle\left\langle\text{sin}^{2}\beta\right\rangle}\cdot\frac{\text{E}_{\text{z}}^{2}}{\text{E}_{\text{y}}^{2}}$$

where *ζ* is the angle by which the plane of the β-sheet tilted to the membrane director and *β* is the tilt angle of β-strand in the plane of β-sheet, expected for a stagger by one residue in the H-bonding between adjacent strands and therefore is consistent with the β-sheet geometry. < cos^2^β > = 0.67 taken from the report by Marsh et al. (1997).

*Φ*, the angle between the β-strands molecular axis direction and the membrane director, is obtained by (Marsh et al., 1997):

$$\text{cos}\Phi\text{ }=\text{ cos}\zeta\cdot\text{cos}\beta$$

***Figure S1. The hypothetical*** ***arrangement of HA2 TMD and FP showing shorter distance between the N-terminus of TMD and the C-terminus of FP than other orientations due to unequal length of TMD and FP***
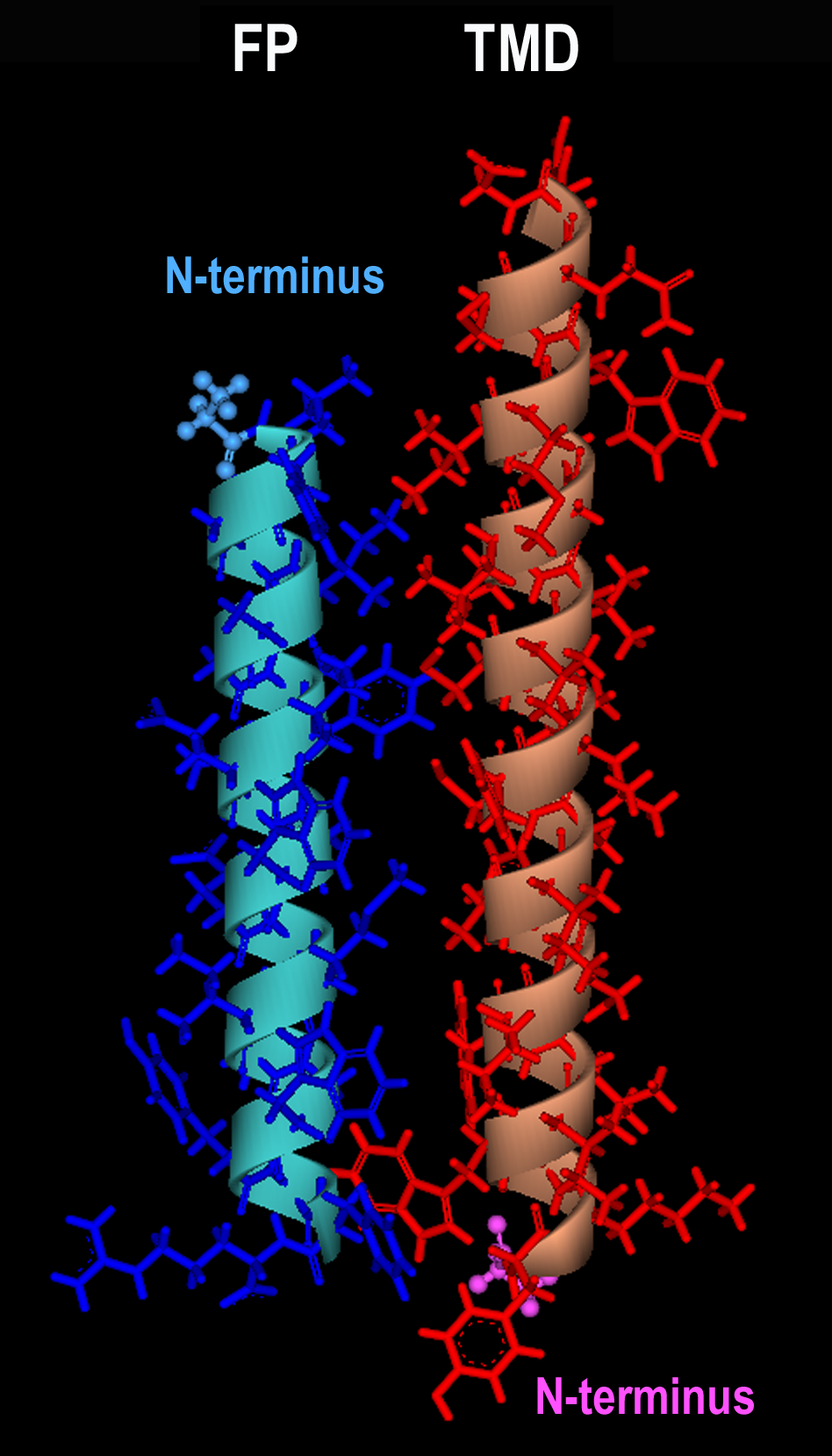


***Figure S2. Deconvolution of IR spectra***

**
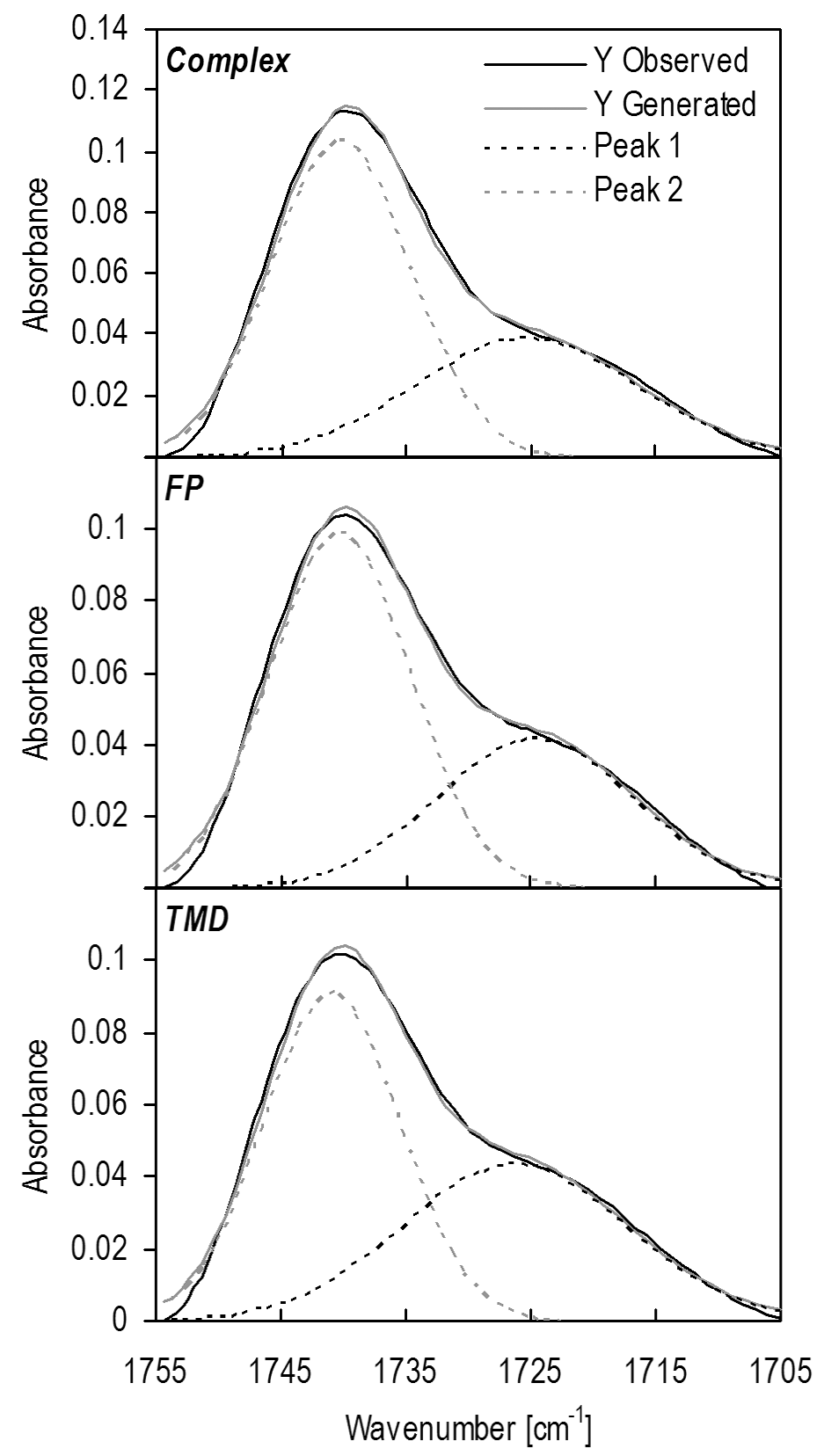
**

**Supplemental references**

Marsh, D. 1997. Dichroic ratios in polarized Fourier transform infrared for nonaxial symmetry of beta-sheet structures, *Biophys. J.* *72*:2710-2718.

Marsh, D., M. Muller, and F.J. Schmitt. 2000. Orientation of the infrared transition moments for an α-helix. *Biophys. J. 78*:2499– 2510.

Harrick, N.J. 1967. Internal Reflection Spectroscopy, Wiley, New York.

Tamm, L.K., and S.A. Tatulian. 1993. Orientation of functional and nonfunctional PTS permease signal sequences in lipid bilayers. A polarized attenuated total reflection infrared study. *Biochemistry* 32:7720-7726.

Tamm, L.K., and S.A. Tatulian, S.A. 1997. Infrared spectroscopy of proteins and peptides in lipid bilayers. *Q. Rev. Biophys. 30*:365-429.
